# An evaluation of a community-based intervention in England aiming to reduce inequalities in exercise participation

**DOI:** 10.3389/fspor.2025.1505249

**Published:** 2025-06-20

**Authors:** Jane Wills, Katya N. Mileva, Susie Sykes, Charles Graham, Chris Flood, Catherine L. Jenkins, Jessica Owugha, Terassa Taylor-Kaveney

**Affiliations:** College of Health and Life Sciences, London South Bank University, London, United Kingdom

**Keywords:** exercise, physical activity, exercise referral scheme, inequalities, evaluation

## Abstract

**Introduction:**

Exercise referral schemes are a common intervention that seek to address physical inactivity. There is limited evidence on whether they can address the inequalities in inactivity associated with income, age, and gender. A novel intervention that sought to address barriers to the uptake of physical activity schemes including access, cost, and specific health and social needs of participants was evaluated in a mixed methods study.

**Methods:**

Serial qualitative interviews with participants were conducted across three time points over the six-month intervention and the routine outcome data collected by the delivery partner were statistically analysed after stratification for referral route, demographic and socioeconomic status and engagement. Data from non-participants in the intervention from the targeted community were collected through street intercept surveys. A descriptive cost analysis was undertaken to understand the cost of delivery.

**Results and discussion:**

The study found small improvements in health outcomes and engagement. The novel features of the intervention that aimed to address inequalities in the uptake of physical activity—personalised programme, extended time offer, free and subsidised offer, a dedicated health coach—all succeeded in acting as enablers to uptake although very few individuals met the recommended frequency for attendance.

## Introduction

Tackling physical inactivity is a global public health priority (1) and there is a clear association between social inequalities and physical activity inequalities particularly in relation to income and gender (2). There is increasing concern in the UK that physical inactivity has become acute across disadvantaged groups and areas of high deprivation. This is particularly the case for women, over-75 s, disabled people and people with long-term conditions and those from minoritised backgrounds (3). Evidence is starting to point out that interventions that target inequalities may have up to four times more potency for reducing disease prevalence than population-wide (universal) approaches (4) and yet according to Oliver et al. (2021) (5), there is limited exploration of common approaches to physical activity promotion (policies or services) through an inequality lens.

Exercise referral schemes (ERS) are an established model for encouraging physical activity whereby those identified as potentially benefitting from being more active (those who are inactive/sedentary and/or living with a health condition) are referred to an exercise programme. There has been a sustained growth of ERS since they were first formally introduced in the 1990s and today around 600 ERS exist across the UK (6). Most ERS schemes use referral from health care professionals, mostly GPs (7) and despite attempts to encourage primary care physical activity pathways for over two decades, little has changed regarding the views of health care professionals on their role in exercise promotion including lack of time, lack of feedback regarding the patients referred, and the belief that physical activity promotion is not a priority during routine health-issue related consultation (8–10).

In line with the NICE recommendations of 2014 to offer ERS just to those with long-term conditions (11), most ERS strategically target those with either poorer health or who are at risk of poorer health (e.g., pre-diabetes, fall prevention) (12). It would be expected then that they would be of greater benefit to those with greater health needs. The evidence however, on whether an ERS can help to reduce inequalities in physical activity, is equivocal. A recent analysis of referral, uptake, and adherence to the Welsh national ERS over the period 2008–2017 found that those in more deprived areas had lower uptake with a decrease over time in referrals and uptake rates compared to those in the least deprived group (6). Other evidence is less clear cut: increasing deprivation was found to be associated with greater adherence in one large scheme (13) but another study of over 300 general practice surgeries found no relationship between deprivation and uptake or activity although GPs within areas of deprivation were more likely to refer patients (14).

There is a substantial body of qualitative data highlighting the psychosocial factors that influence uptake and adherence in ERS that include intrinsic motivation, psychological needs satisfaction, and self-efficacy (15). Recommendations from a literature synthesis about what needs to be in place for individuals to be motivated and take up activity offers include social and psychological support, role models and peer-to-peer influencing to foster self-efficacy (16, 17). It is also known that the offer needs to be both fluid and flexible to meet practical, environmental, social, and psychological individual needs and to be freely available and accessible (16, 18).

A range of key issues and suggestions focussing on increasing the uptake of ERS became evident from the numerous publications on their effectiveness since emerging in 1990s. This paper reports on the evaluation of a physical activity intervention which attempted to incorporate these recommendations in, for example, signposting pathways, not using the term “exercise” but rather focusing on health and wellbeing (19) and personalised interventions (20). This evaluation study adds to the extensive body of literature on ERS by reporting on a novel intervention designed to address known barriers through an equity lens, offering insights into how such schemes can be structured to reduce inequalities in physical activity uptake, particularly among disadvantaged and underserved populations.

The intervention incorporated various novel features to address the known barriers to physical activity. These novel features included that although the intervention was available to all, it was made free of charge to those in receipt of social benefits and targeted at people from disadvantaged communities and those with protected characteristics, but others could pay for the intervention. This approach aligns with the current emphasis in public health strategy on proportionate universalism (21) whereby services are made universally available but targeted to and responsive to those in greatest need. Secondly, it was intended that referrals would be made not only from health professionals as other schemes but also from social prescribers, the voluntary sector and via self-referral (22). Thirdly, the offer was that following an initial assessment with a health and well-being physical activity coach, participants would receive a personalised bespoke exercise programme to include one-to-one or small group work and activities based in a leisure centre or community setting and not just a designed rehabilitation programme (12). Fourthly, the intervention was intended to last six months, which is longer than the usual ERS duration of 12 weeks (23). Lastly, to reflect its ambition to address the determinants of physical inactivity and their association with deprivation, the dedicated health and well-being coaches were also expected to refer to other community services when needs related to health are identified and addressed e.g., housing, debt, loneliness.

The intervention was developed and offered in a borough in the UK where the levels of deprivation are relatively low with just under half of neighbourhoods in the least deprived 20% of the country. However, 16% of its neighbourhoods are considered more deprived than the national average. The majority of the population identify as White British (78%).

This paper reports on a process evaluation for the novel intervention with the aims of identifying its reach, how well it was implemented and whether it was being taken up by the targeted underserved populations. A second aim was to investigate whether what is delivered locally enabled the achievement of the mechanisms of causation described in the intervention's theory of change and the health and wellbeing outcomes of participants. As well as reporting participant outcomes, we report on the process in accordance with the recommendations of the Medical Research Council (24) to better understand how the local context may have influenced outcomes, and to provide insights to aid implementation in other contexts.

## Methods

### Theoretical underpinning

Following guidance of the Medical Research Council (MRC) (24) this evaluation adopts a programme theory that aims to understand how and under what circumstances interventions lead to change. Not only is the focus on whether the intervention is effective in encouraging physical activity amongst the sedentary populations, but also on how it might do this. Theory-based evaluation relies on a programme theory explaining how an intervention is expected to generate its intended outcomes. Creating a logic model is an initial stage of evaluation which helps to map out the underlying assumptions and theories of change on the factors that drive its effectiveness This took place through three coproduction workshops facilitated by the evaluation team with participation from local stakeholders, and public contributors [Public and Patient Involvement and Engagement (PPIE) representatives] to formulate these short, medium and longer term outcomes about how the novel features outlined would encourage more uptake of the physical activity programme by sedentary populations and those most in need. The following assumptions underpinning the intervention design were identified (25):

a) If participants are given a one-to-one appointment with a coach during which a co-created plan is created, then there is a greater chance of a sustained change in PA because the intervention is tailored to them, so, they are more likely to commit.
b) If participants can choose from a variety of forms of exercise and are offered flexibility, including the option for social interaction, then adherence will improve because they have more personal choice and enjoyment.
c) If participants in deprived areas are directly offered the intervention, then they are more likely to participate because it has no cost and there will be fewer barriers to access.
d) If all referrers (primary care, social prescribing and voluntary sector) have a clear understanding of the nature and aims of the intervention, have some training, have clear, accessible guidance and there are processes to support referral, then they will participate more and refer more appropriately.
e) If all practitioners involved with a potential user have a clear understanding of the intervention and other partners continuity will improve, people referred will be suitable, and inter-professional/inter-sectoral cooperation and mutual value will improve.

### Study design

**Figure 1:**
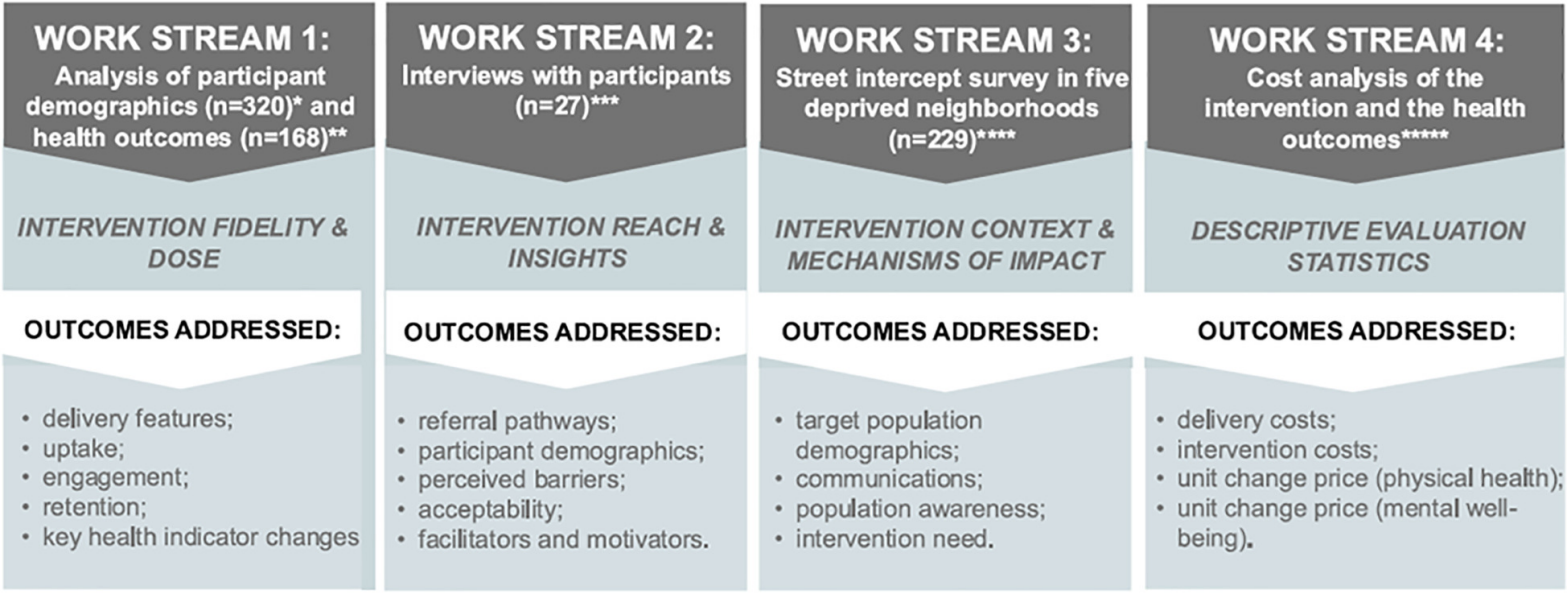
Study design and evaluation components for the monitoring phase of the conducted process evaluation. The assessment focused on the ability of the community-based exercise referral intervention to address inequalities in physical activity uptake. The four workstreams and the outcomes addressed are aligned to the key components of a process evaluation according to the MRC's framework for developing and evaluating complex interventions (24). *(*n* = 320) – number of individuals referred to the intervention from 1st November 2022 to 31st October 2023; * (*n* = 168) – number of ‘referred engaged’ individuals; ***(*n* = 27) – number of interviewed referred individuals; ****(*n* = 229) – number of interviewed ward residents from the target population; ***** - cost analysis of each health outcome change (pre- vs. post-intervention) based on the number of complete cases (*n* = 9-to-104).

The co-production workshops led to an agreed evaluation design comprising four work streams to investigate how the specific characteristics of the intervention (e.g., cost, length, referral routes, personalised programme) influence uptake, engagement, acceptability and impact on participants and target populations. In line with MRC guidance (24) which stresses that purely quantitative or experimental approaches are rarely adequate for complex intervention evaluation, a mixed methods approach was adopted. The study consisted of four workstreams (Figure 1) that addressed outcomes such as: reach, recruitment, engagement, adherence, changes in key health outcomes (work stream 1; analysis of participant data (*n* = 320) reports on intervention dose delivered, dose received in exposure and reasons for uptake, and fidelity of the intervention components; workstream 2; individual interviews with participants (*n* = 27) reports on completeness and reasons for adherence, satisfaction; workstream 3 through street intercept interviews (*n* = 229) sought to understand the context for delivery and identify barriers and facilitators including community awareness; workstream 4 was a descriptive cost analysis of the intervention delivery and the key health outcomes.

All recruitment and data collection and reporting methods were carried out in accordance with the Declaration of Helsinki. Ethical approval was secured from London South Bank University Ethics Committee ETH2223-0240 and ETH2223-0234 and carried out in accordance with this. All participants provided formal written or recorded verbal informed consent to participate.

### Participants

The first workstream used anonymised survey data of 320 individuals referred to the intervention from 1st November 2022 to 31st October 2023 that was collected by the intervention deliverers via the JOY system (26), a digital system for accessing non-clinical support services.

The second workstream employed qualitative semi-structured serial interviews (*n* = 27) (27) with participants who agreed to be approached by the research team. Intervention participants were invited to be interviewed at three different time points over the six-month programme: soon after referral, mid-way through the six months and at the intended end point of participation.

The third workstream comprised a cross-sectional street intercept face-to-face survey in the five most deprived neighbourhoods of the borough. The timing, late in the research process, allowed for any type of communications affecting awareness of the intervention to diffuse across the population. To identify sample elements, participants were screened as (i) resident in a target ward, (ii) inactive as defined by International Physical Activity Questionnaire (IPAQ) (28) (iii) aged over 18 years and (iv) a non-user of the intervention.

### Data collection and analysis

The JOY system generates real-time health outcome data [e.g., health and wellbeing improvement including Patient Health Questionnaire (PHQ –9 Depression); resting heart rate; body weight; Body Mass Index (BMI); waist circumference; blood pressure; and Short Warwick-Edinburgh Mental Wellbeing Scale (WEMWBS)). The population average health indicators were descriptively characterised and statistically compared between the entry and the latest measurement points. These data were also analysed with consideration of the demographic data collected by the JOY system to explore the impact of the novel features on uptake and adherence by deprivation level, age, ethnicity, and health status.

Each qualitative interview conducted with the intervention participants as part of the 2nd workstream lasted up to an hour and took place during a 10 month window. Interviews were not intended to measure change over time but rather to explore the participants' experiences of the specific features of the intervention, barriers and facilitators to uptake and self-reported outcomes at different stages of the intervention. Data were analysed thematically with a combined inductive and deductive coding approach and supported by Delve analytics software (29).

The street intercept survey explored awareness of the intervention, knowledge of local opportunities for exercising, and any engagement with physical activity. Surveys were conducted in four local retail locations and participants were given a £10 voucher on completion of the interview survey. Data analysis included thematic analysis of open responses and frequency analysis of the multiple choice questions.

The fourth work stream comprised a descriptive cost evaluation of the intervention delivery and the changes in health outcomes per participant and per unit of change. A full economic evaluation was not possible due to the limitations of the data collected and the timing of the evaluation against intervention delivery. The unit costs of delivering the intervention were identified and included staff time (i.e., all staff involved, their hourly rates and total number of hours involved in the intervention, the total budget allocated to the intervention and the cost of the actual intervention (disaggregated and described separately to the overall budgetary costs), any additional training costs, any additional building rental, office space or equipment cost as well as additional IT and travel costs. These data were analysed alongside service user patient monitoring data.

## Results

The intervention was originally designed to have various features that are novel and distinguish it from other exercise referral schemes. Most of these were incorporated but the vision for physical activity to be part of a holistic approach to wellbeing and health improvement was not realised. The referral routes to the programme were not as extensive as intended and came primarily for social prescribers situated in GP practices or self-referral. The intention that the intervention would offer flexibility and a variety of activities to meet needs was not implemented and the intervention was confined to a leisure centre operated by a private contractor. The location of an intervention in a leisure centre created barriers of accessibility when there are limited transport links. Similarly, the wellbeing coach did not refer participants to other possible health improvement activities or organisations such as walking groups or mental health agencies.

### Intervention uptake

**Figure 2:**
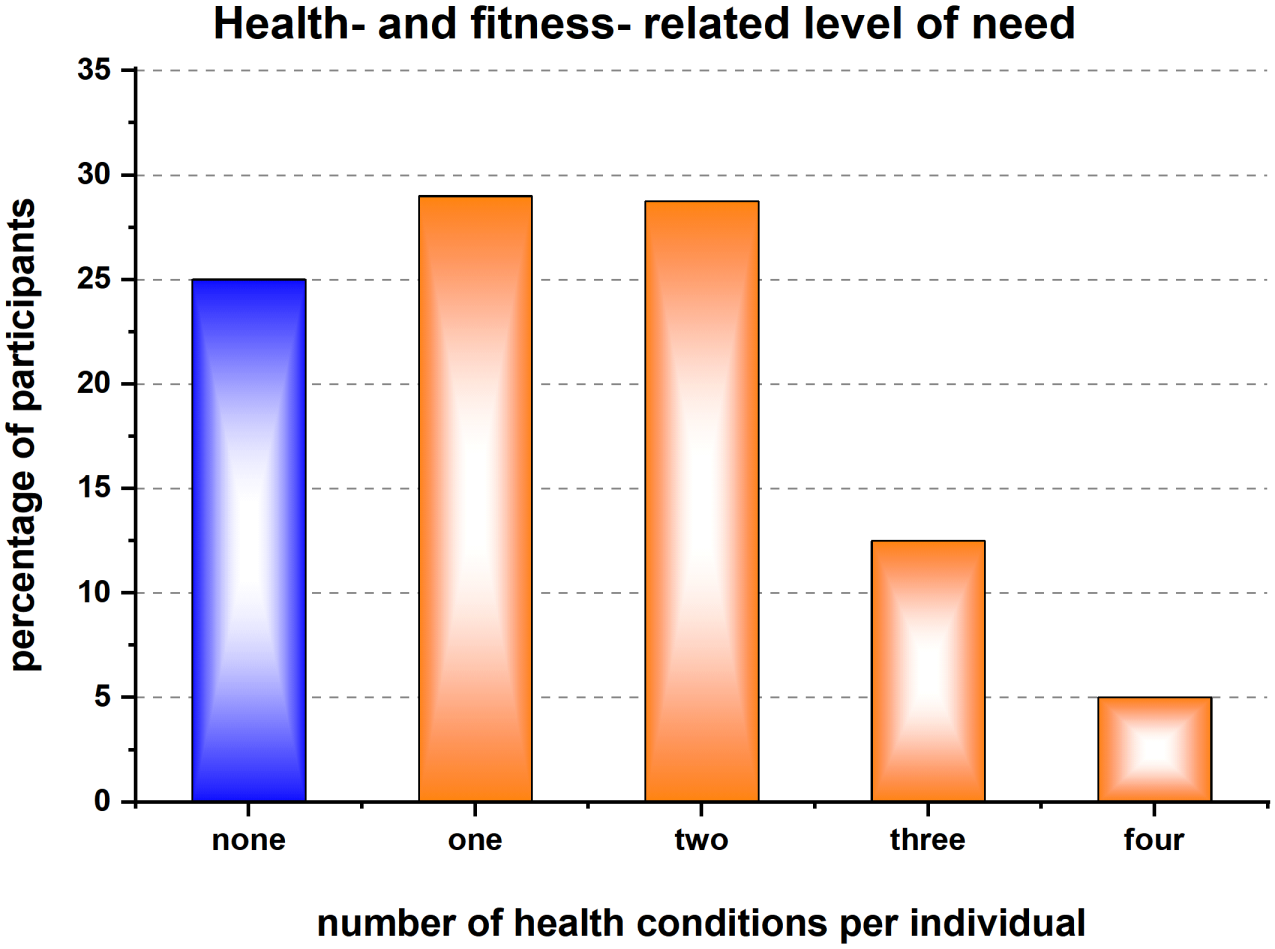
Percentage distribution of the 320 participants according to the number of health conditions, for which they were (self)-referred to the intervention. For 75% of the participants the referral was based on health needs such as obesity, muscular skeletal illnesses (e.g., injury, arthritis, disability or frailty), mental health problems and/or long-term health condition (e.g., respiratory, or cardiovascular, cancer, diabetes, and other noncommunicable diseases.

The intervention was targeted at those from economically deprived neighbourhoods and those with long term conditions who are more likely to be physically inactive. It was partly successful at reaching these target groups. 320 individuals had been referred to the intervention in the year, of whom 40% were residents of the five most deprived wards and 42% were eligible for free use i.e., in receipt of benefits. According to recent data from the local council's strategic planning document, only (10% of the area residents are low-income households (30), which may have bearing on the findings. Most intervention participants identified as female (59%), the average age was 51 years, and ethnicity was not recorded for a large proportion of the participants. Figure 2 shows that 75% of those referred to the intervention had identified health needs and 46% were registered as having more than one (up to four) health issues. More specifically, 39% of intervention participants were registered with at least one long-term health condition (e.g., respiratory, or cardiovascular, cancer, diabetes, and other noncommunicable diseases), 18% had a muscular skeletal illness including injury, arthritis, disability or frailty, 31% had a mental health problem and 32% were referred for an obesity or weight management issue.

Of the 320 referred individuals over two years, 173 (69%) had been referred from a primary care network (PCN) of which there are five in the area. Established by NHS since 2019, the PCNs group several general practices (GP) together and other health and care providers with the intent to facilitate a wider range of service delivery to the local population. The PCNs level of engagement varied by referring between 3% and 36% of the participants in the intervention. Referring agents from 11 general practices were identified. Additionally, 15% of participants came via self-referrals and further 11% joined the intervention on referrals from one of the four local acute hospital trusts that are providing secondary care to patients and are getting widespread in the UK (13).

Street intercept surveys were conducted with 229 non-participants in the intervention in four retail locations in the most deprived neighbourhoods in the borough and the characteristics of this sample match those of the population in that area weighted for inactivity and reported age, ethnicity, receipt of benefits and health status of the target population. 80 of these participants (35%) had been advised by a health care professional to be more active and 135 (59%) had seen a GP in the previous six months but nevertheless had not been referred to the intervention. 2% of the non-participants had at least one health condition, 20% suffered anxiety depression or stress and 15% hypertension.

### Intervention engagement

**Figure 3:**
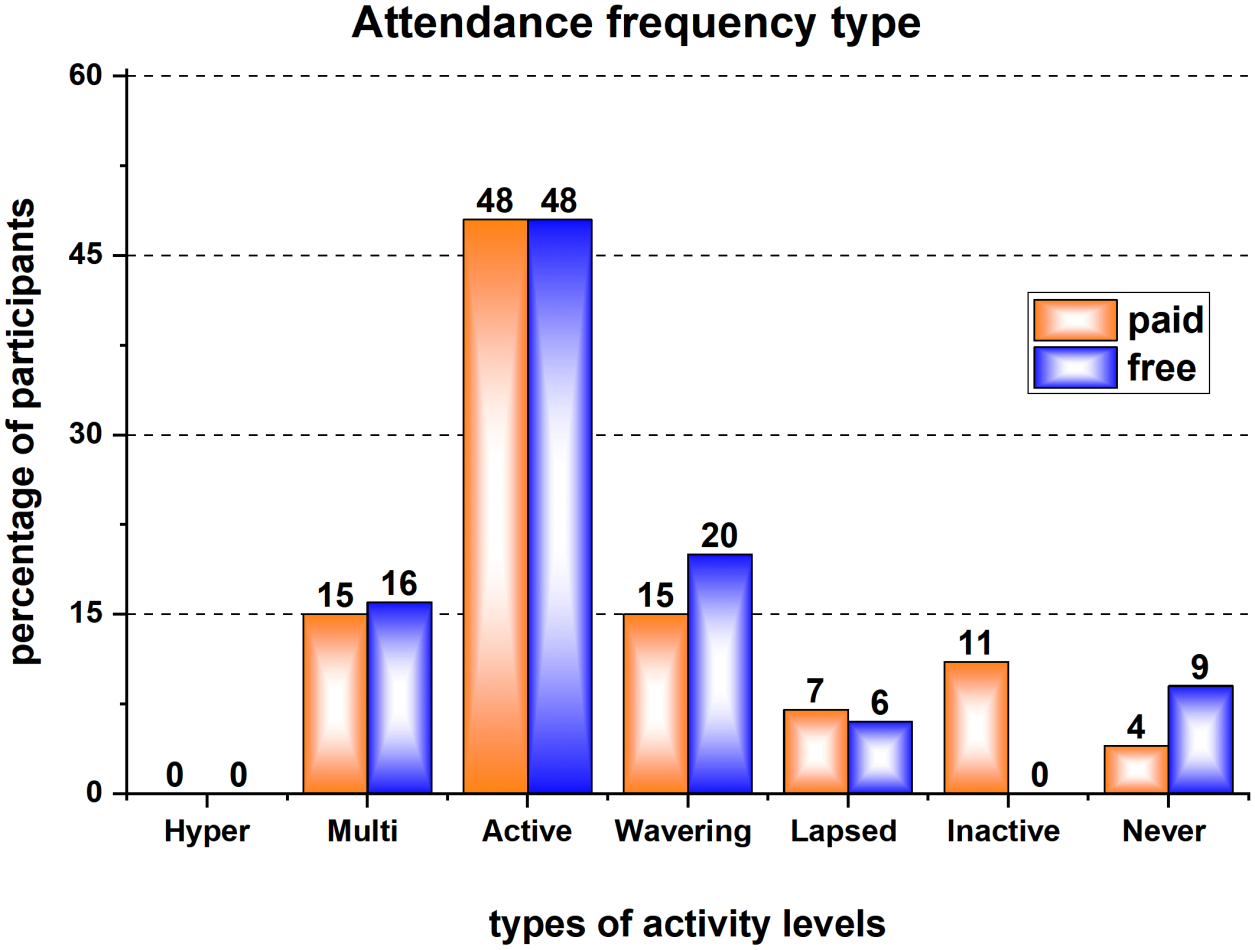
Percentage distribution of the 320 participants into 7 groups according to the frequency of their attendance to the intervention sessions: “Hyper-Active” - attended 8 + times in every month for the last 6 months; “Multi-Active” - attended 8 + times in the last month; “Active” - attended 1-7 times in the last month; “Wavering” – no attendance in the last month but attended in either of the previous months since registration; “Lapsed” – no attendance in the last 3 months but have attended in the last 6 months; “Inactive” – no attendance in the last 6 months; “Never Active” – no record of any attendance since registration.

The recommended physical activity guidelines are 150 min per week and for this intervention this equated to attending at least two sessions per week. As can be seen in Figure 3 very few of the participants were active and reaching this recommended “dose”. The referred 320 individuals were classified into seven clusters according to their monthly frequency of attendance to the intervention sessions. Active were only ∼1 in 6 (15% of those who paid to attend and 16% of those receiving the intervention “free”) individuals who achieved the recommended attendance frequency of ≥2 per week for six months (Figure 3). 48% from each category (“paid” and “free”) were classified as active and visited the gym between 1 and 7 times in a month. The duration of the visits (active time) and the type and intensity of undertaken activity/ies were not recorded.

The intervention was intended to address some of the known inequalities in physical activity and the take up of exercise referral schemes. Interviews were conducted with 27 intervention participants, 13 completing all three interviews, eight completing two interviews and six completing just the first interview. The interviews revealed that intervention participants typically had complex lives and therefore mixed motivations for undertaking physical activity. Several had a series of health conditions that required hospital stays or a complex schedule of appointments to attend. They also had mental health and well-being issues alongside these physical health conditions, and many had multiple caring responsibilities resulting in repeat interruptions to their physical activity:

“Because it just had been at the wrong time and I just couldn't throw myself into it. Because, obviously, COVID; I was ill as well, moving house, my step-dad, my head, my therapy: everything's just happening at once and, on a day-to-day, I just struggle to even, get through the day.” (Participant 23 Interview 3).

The intended facilitators of free or low cost, extended time period of engagement and a personalised programme with a dedicated coach all were reported as helpful.

(a) Cost

The free and subsidised offer was seen as a strong motivator, and affordability was a serious concern with most saying that they would not continue with the intervention if the subsidy or free offer ended after six months:

“Obviously, I was in the bracket where all of it was free. And, yes, I wouldn’t go or have been able to go or even to step in that place without it.” (Participant 23 Interview3)

(b) Time frame

The extended six-month timeframe of the intervention was highly valued by those participating in it. The complexity of their lives, as well as the need to manage complex health conditions which often involved treatment, meant there was often a need for people to pause their involvement.

“So, you need a length of time for you to start to feel better from the hard work, because it is hard work to start. You need a longer time period for people like myself for it to go from being a real arduous task to a pleasure to go there and feel good about yourself and six weeks is far too short, or it was for me anyway.” (Participant 21, Interview 3)

(c) Personalised programme

Tailoring and personalisation were seen as a strong motivator but undertaken within the leisure centre itself with intervention participants directed to different physical activity opportunities (primarily the gym and swimming):

“It was great that it was six months but I still really appreciate the fact that he took me round the gym the first time and he sort of found out what my illnesses were, what my pains were and tailored it for me and went round with me and showed me everything and without that I probably wouldn’t have been confident to get on some of the equipment or even go up to ask, but I felt comfortable then because he came round with me in the gym.” (Participant 17 Interview 3)

The role of a health coach is a crucial enabler to the intervention and key to engagement and regular communication providing support. This demanded a sophisticated skills set and understanding of the complex barriers and health and well-being conditions facing the target groups.

“….because you had somebody to talk to about it who understood what you were doing and why you were doing it, they were also able to give you some guidance on how to do it and you also felt you were committed to a contract with them.” (Participant 21, Interview 3)

### Health and wellbeing outcomes

Those completing the six-month intervention reported modest but important shifts in health, well-being and lifestyle including feeling happier, increased confidence, resilience and reduced social isolation based on the short form of the WEMWBS. There were improvements in self-reported physical health and fitness but for some of the intervention participants these were quite small; there were limited reports of weight loss, but some changes in eating habits (see Table 1). For some intervention participants, exercise had become routinised and most intended to continue some form of exercise and try to increase the amount of physical activity:

**Table 1:** Health measures (mean ± standard deviation, SD) taken by the health coach at the start (baseline), after 3-months (follow-up) and 6-months (completion) of the intervention from the two main groups of participants: (i) discharged - who by the time of this evaluation had completed the full 6-month exercise programme, and (ii) active - who entered the intervention later and were yet to complete the full 6-month programme. Negative or positive value of the percentage change indicates reduction or increase, respectively, of the measure. The interpretation of this direction of change is measure-specific and transcribed in the legend below.

Health measure		Discharged—completed the 6-month intervention (*n* = 19); mean ± SD			Active—continuing the 6-month intervention (*n* = 149); mean ± SD		
		Baseline	Completion	% change	Baseline	Follow-up	% change
Mental health	Patient Health Questionnaire (PHQ-9), score	14.0 ± 0.0	4.5 ± 3.5	−67.9%	16.8 ± 7.8	12.4 ± 5.2	−25.8%
	Short Warwick-Edinburgh Mental Wellbeing Scale (sWEMWBS), score	51.1 ± 20.3	64.3 ± 19.1	+25.9%	54.8 ± 17.8	62.2 ± 26.2	+13.5%
Physical health and fitness	Waist Circumference, cm	113.1 ± 18.7	109.8 ± 17.6	−2.9%	113.0 ± 14.7	114.2 ± 15.0	+1.0%
	Resting Heart Rate (rHR), bpm	83.3 ± 14.5	79.8 ± 14.8	−4.2%	80.3 ± 11.6	81.5 ± 13.2	+1.5%
	Body Weight (BW), kg	96.4 ± 30.7	94.9 ± 29.9	−1.5%	101.2 ± 21.6	103.3 ± 20.2	+2.1%
	Body mass index (BMI), kg/cm^2^	35.3 ± 8.5	34.8 ± 8.3	−1.4%	36.7 ± 8.1	36.8 ± 6.5	+0.3%
	Systolic Blood Pressure (sBP), mmHg	133.0 ± 21.5	125.8 ± 20.4	−5.4%	133.7 ± 21.3	133.4 ± 19.6	−0.2%

PHQ-9 (Depression severity scale): none (0÷4); mild (5÷9); moderate (10÷14); moderately severe (15÷19); severe (20÷27). sWEMWBS (mental wellbeing): range between 7 and 35; the higher the score the more positive wellbeing. rHR (cardiorespiratory function): age, sex and fitness-specific; a lower rHR implies more efficient heart function and better fitness. BMI *(obesity index):* underweight (15÷19.0); normal (20÷25); overweight (25÷29.9); class I obesity (30÷34.9); class II obesity (35÷39.9); class III obesity (≥40).

“I’ve noticed, now, when I take the dog for a walk, I’m not struggling as much as I was before. I would get out of breath really quickly.” (Participant 16 Interview 3)

“I’ve got into the habit, at the same time, practically every day, that I will go to the garage and now I will go to the gym—particularly recently. So that's like, it's in my routine”. (Participant 26 Interview3)

### Cost of the intervention

A simple cost analysis included in this evaluation of this intervention found the cost per participant was £172.40 (all costs are reported in Pound Sterling). An overall average cost per change in improvement for the intervention (where this occurred) was calculated for each variable of interest: mental wellbeing (−£28.73); resting heart rate (−£188.73), weight in kg (−£147.29); blood pressure (systolic)(−£32.52); BMI (−£294.34); waist circumference (−£274.76). There was a higher cost per unit of improvement for all outcomes for intervention participants (whose health was poorer) who were offered a free intervention when referred by a health care professional. This analysis is limited by a smaller sample available to evaluate referral source data.

## Discussion

Reaching inactive populations who have protected characteristics and/or live in areas of deprivation is clearly a priority in most countries. Numerous evaluations of ERS have sought to understand what factors influence uptake and completion of a programme finding that age (31) and a cardiovascular condition (10) are the most consistent predictors. This intervention was designed to offer tailored support from a coach, a variety of activities, be free or low cost and to be made available to those who would most benefit through multiple referral routes in the community.

This paper focuses on a novel intervention designed to address known barriers through an equity lens, offering insights into how such schemes can be structured to reduce inequalities in physical activity uptake, particularly among disadvantaged and underserved populations. Synthesis of the present findings and those from the extensive body of literature on ERS underpins several recommendations to effectively tackle physical inactivity and the inequalities in physical activity uptake:

### Expanding and diversifying the referral pathways

Most of the participants of the intervention in this study were referred by a health care professional but not all general practices were active referrers. The most deprived and least well attend health services frequently and yet awareness of the intervention in the target deprived wards was very low. Most of the non-participants in this study had never seen publicity about the intervention and so self-referral (32) was unlikely; referral routes for such an intervention would be most likely to come from GPs and other healthcare professionals, and yet this was not happening and a missed opportunity. Many of those in the deprived areas would like to be more active, with motivations including improved health and avoidance of ill health, but were not aware, or had not been identified, as potential participants for the intervention. This intervention had the intention of diversifying referral routes using a wide range of frontline agencies such as housing offices, benefits offices and mental health services. This did not happen due to a lack of co-ordinated awareness raising and because the provider was the single leisure service. This echoes a recent study of referral routes that identifies the need for closer working with link workers and navigators (33) and initiatives to increase the role of social prescribers (22). The role of social prescribers does vary: those attached to general practices worked closely with GPs and advanced nurse practitioners and recognised the potential of physical activity in contributing to mental health and weight loss. Social prescribers employed in the community worked with a caseload and as more a support worker with clients who were not able to participate in such a programme either because of serious mental illness or because they were recently discharged from health care.

### Adopting personalised approach to uptake and engagement

This study found that uptake of the physical activity intervention is greatest among women and those aged under-55 and the intervention is less successful in reaching ethnic minority communities and the most sedentary. Those who were referred into the intervention had complex lives with multiple caring responsibilities and had a combination of physical and mental health conditions for which they were receiving treatment. This created multiple barriers to individuals' attendance. In line with evidence from other services (31), very few participants met the recommended frequency for attendance (Figure 3) and most of those referred either declined participation (20%) or did not engage regularly (13%) or left early (4%). A personalised approach has been frequently cited as a facilitator of adherence (34).

### Implementing a whole system approach to promotion and delivery

There are entrenched and complex social, political and economic determinants of physical activity and change is required at individual and collective levels. In public health practice, there have been several national-level initiatives to promote systems thinking in addressing complex public health issues such as physical activity and obesity (35). This intervention was intended to be part of a wider system of physical activity promotion whereby it would include a range of activities and referral routes from agencies and organisations in the community including the “diversification” of the large number of sports clubs towards the inactive (36). This was not implemented, and the private provider confined the intervention to the leisure centre thus restricting uptake and adherence to those with confidence, social support, and funds to travel to and from the facility.

### Associated mental as well as physical health benefits from ERS

The evidence from this study, albeit from a small number of intervention participants who completed the full six-month intervention, is that there are discrete but measurable benefits across both physical and mental indicators of health. For many participants, their mental health was an important reason for joining the intervention and making connections and reduced isolation were outcomes hoped for. Mental wellbeing improved and the costs for this was the least of all outcome measures, demonstrating that physical activity and mental health and wellbeing should be thought of in less siloed ways when developing interventions to address sedentary behaviours and inactive populations (37). Improvements in physical health were small with some slight improvement in cardiorespiratory function and a few participants had a slight weight loss. The majority of the participants had pre-existing health conditions and results vary reflecting the reasons for referral and individual characteristics. This intervention shows only limited effect in improving health indicators which reflects the equivocal evidence about such schemes over the last two decades (11).

### Addressing barriers to uptake, adherence and retention

This intervention sought to address barriers identified in other studies to the uptake of physical activity offers such as cost and support (16). In this study, cost was found to be a relatively lower obstacle to uptake of physical activity than time although cost was a motivating attraction. The free and subsidised offer included in the intervention was a key enabler both for those who took up the intervention and those who would be in the targeted group but hadn't been referred. This is in line with some other evidence (18) although an evaluation of referrals to the Welsh national ERS found an increase in price in 2011 in the cost/session from £1.00 to £1.50 was followed by a rapid falling in attendance among people in the most deprived groups (6). In this study the cost that would be incurred beyond the six-month period did present a barrier to continuation for some but there was little difference in engagement and adherence between those who paid for the intervention and those who received it free.

### Scaling-up to enhance cost-effectiveness

The costs of physical inactivity globally are now being calculated albeit with the caveat that few studies identify the separate cost benefit of avoiding inactivity-related diseases such as coronary heart disease, stroke, type 2 diabetes, hypertension, cancer (breast, colon, bladder, endometrial, oesophageal, gastric, and renal), dementia, and depression (38) which, if successful, results in an ageing population and other associated healthcare costs. In the UK, a guidance document (16) identifies physical inactivity as associated with 1 in 6 deaths in the UK and is estimated to cost the UK £7.4 billion annually (including £0.9 billion to the NHS alone). The cost of inaction on physical activity for healthcare systems is highlighted in a report for WHO in The Lancet (39). Various studies have conducted cost effectiveness analyses and have generally concluded that ERS are not economically viable (10) but based on data that is frequently incomplete and limited in size and scope. This study is similarly small, and the outcomes are modest, but the in-depth interviews with participants show an interest and motivation in reducing inactivity and seeing its benefits. The costs of this intervention (average £172.40) that sought to address inequalities would be reduced by scaling up the intervention with continued and focused targeting of the most inactive and deprived groups who also showed an interest in being more active to benefit their health but who were not being reached.

There was an ambition among those completing the intervention that they would continue to maintain physical activity. This study did show however, the persistent social gradient that those most in need of offers for exercise participation were not reached and those who benefitted most from the intervention were those who self-referred and/or paid for the intervention. The higher costs per outcome for those referred to the free intervention is not a surprising finding but does show that targeting inequalities in physical activity requires a higher investment.

### Limitations

This is a study of just one intervention and over the period only a small number of intervention participants completed the full six-month intervention during the data collection window, so reducing the sample size and the power of analysis. The software used for data collection (JOY) was not customised for the intervention and did not allow full data export for evaluation purposes. JOY also proved incompatible with assistance routinely built by the delivery agents. This limitation of the recording and monitoring of physical activity schemes is one that has been previously identified (5). People from the Asian, Black and Other ethnic groups are more likely than average to be physically inactive, at 31%, 29%, and 30% respectively but ethnicity was not recorded on the system and so we are unable to provide important insight into whether the intervention addresses the particular barriers experienced by people of ethnicity. The intervention was delivered in a relatively affluent locality but other factors of age, gender and existing health conditions were additional factors explaining the inequalities in exercise participation and the concluding recommendations are likely to apply across different contexts. The descriptive cost analysis provides useful preliminary insights, but there are important limitations including the small sample who completed the programme, some missing referral source data and the only modest health outcomes. We cannot claim the cost effectiveness of this intervention but we do point to its relatively low outlay and the reported improvements in mental health.

## Conclusions

A report from Public Health England (2021) identified that if inequalities in physical activity uptake are to be addressed, there should be full engagement with the community to ensure that all interventions all are needs-driven and that the needs of those with protected characteristics are addressed (17). This study found that any physical activity intervention needs to be accessible to those with limited access to transport; flexible with diverse opportunities; free or subsidised as this acts as a motivator; use various referring agents as supporters and part of the offer, not just as navigators; and provide opportunities for individuals to connect.

An intervention such as this has the potential to address inequalities in levels of activity and engagement with existing offers, but it needs to be part of wider system strategies for health and wellbeing such as those that include transport and environment, obesity or mental health. Evaluation of its success or otherwise needs to move beyond traditional physical health indicators and reflect the benefits that participants themselves identify as important from activity interventions, such as being with others and structure.

## Data Availability

The anonymised raw data supporting the conclusions of this article will be made available by the authors, without undue reservation.
